# Audit of the medical staffing of and referrals to a mixed adult ICU in a teaching hospital

**DOI:** 10.1186/cc12424

**Published:** 2013-03-19

**Authors:** A Needham, T Lyndham, D O'Neil, J Greiff

**Affiliations:** 1 University Hospitals of Leicester, UK

## Introduction

Leicester Royal Infirmary is a (funded) 15-bed ICU, annual admission 1,102 patients per year (ICNARC data 2011 to 2012). It houses a broad range of specialty, has over 1,000 beds and an emergency department (ED) responsible for 297 admissions over the 2011 to 2012 period. Unit guidelines state that a doctor should be immediately available to the unit at all times, ideally physically present on the unit. This audit looked in detail at each occasion a junior doctor left the unit, the timings, reason, and outcome of each episode.

## Methods

Between 17 October 2011 and 14 November 2011, and between 2 July 2012 and 30 July 2012, junior doctors on the ICU completed a data-capture form after each occasion that they left the unit for a clinical need. Data collected were matched to the objectives. The 2011 audit results were communicated at the University Hospitals of Leicester Trust ICU Audit Meeting. A system was introduced so that daystime referrals were directed to the ICU consultant. The impact of this intervention was assessed by the 2012 re-audit.

## Results

There were 105 occasions when a junior doctor left the unit in 2011, totalling 118 hours and 38 minutes (5 days), a continuation of the upward trend from the previous 2 years. In 2012 this reduced to 47 occasions totalling 40 hours and 55 minutes. Most attended referrals were during the daystime, 54% in 2011 and 68% in 2012. The majority of referrals originated from the ED/medicine, combined numbers 58% in 2011 and 38% in 2012. In 2011 only 30% of ED referrals had been discussed with a consultant of the referring team and only 32% of medical referrals. Just two of the 14 inappropriate ED referrals were discussed with the referring consultant, and none of the six inappropriate medical referrals were discussed with a medical consultant. In 2012 just 33% of ED/medical referrals had prior discussion with a consultant from the referring team. At the point of leaving, the ICU was left without a doctor on 22 occasions in 2011 and six occasions in the 2012 audit. Most occasions occurred at either night shift/weekend (86% in 2011/100% in 2012). Referral resulted in a patient being admitted to the ICU on 20 occasions in 2011 and 12 in 2012.

## Conclusion

The latest audit follows introduction of a referral system directly to the ICU consultant and may account for the reduction in numbers of referrals attended by junior doctors. ED/medicine persist as the main source of referral to the ICU. Discussion with the referring team consultant may reduce inappropriate referrals. ICU staffing should not be reduced.

